# Watch Out for the “Living Dead”: Cell-Free Enzymes and Their Fate

**DOI:** 10.3389/fmicb.2017.02438

**Published:** 2018-01-04

**Authors:** Federico Baltar

**Affiliations:** ^1^Department of Marine Science, University of Otago, Dunedin, New Zealand; ^2^NIWA/University of Otago Research Centre for Oceanography, Dunedin, New Zealand

**Keywords:** marine biogeochemical cycling, carbon cycle, organic matter hydrolysis, extracellular enzymatic activity, cell-free enzymes, climate change, warming

## Abstract

Microbes are the engines driving biogeochemical cycles. Microbial extracellular enzymatic activities (EEAs) are the “gatekeepers” of the carbon cycle. The total EEA is the sum of cell-bound (i.e., cell-attached), and dissolved (i.e., cell-free) enzyme activities. Cell-free enzymes make up a substantial proportion (up to 100%) of the total marine EEA. Although we are learning more about how microbial diversity and function (including total EEA) will be affected by environmental changes, little is known about what factors control the importance of the abundant cell-free enzymes. Since cell-attached EEAs are linked to the cell, their fate will likely be linked to the factors controlling the cell’s fate. In contrast, cell-free enzymes belong to a kind of “living dead” realm because they are not attached to a living cell but still are able to perform their function away from the cell; and as such, the factors controlling their activity and fate might differ from those affecting cell-attached enzymes. This article aims to place cell-free EEA into the wider context of hydrolysis of organic matter, deal with recent studies assessing what controls the production, activity and lifetime of cell-free EEA, and what their fate might be in response to environmental stressors. This perspective article advocates the need to go “beyond the living things,” studying the response of cells/organisms to different stressors, but also to study cell-free enzymes, in order to fully constrain the future and evolution of marine biogeochemical cycles.

## Importance of Microbes and Their Extracellular Enzymatic Activities (EEA)

The marine environment plays a critical role in global biogeochemical cycles (Daily, 2003; Harley et al., 2006; Hutchins and Fu, 2017). Microbes are the engines driving Earth’s biogeochemical cycles (Falkowski et al., 2008). These tiny organisms have the set of core genes coding for the enzymes of the major reactions responsible for transforming energy and matter into (usable) substrates essential for life (Falkowski et al., 2008). We live in a time of change, and anthropogenic impacts can alter the structure and functioning of marine microbial communities, and consequentially, the role of the ocean in the global biogeochemical cycles. Thus, if we aim to understand what the future of marine biogeochemical cycling is going to be, we need to understand what the fate of microbes, and their enzymes, will be.

When it comes to the consumption of organic matter for transformation and recycling, microbes seem to have a preference for specific types of organic matter. According to the “size-reactivity” model, heterotrophic microbes preferentially degrade high molecular weight dissolved organic matter (DOM) because it tends to be more bioavailable than the low molecular weight DOM (Benner and Amon, 2015). But, that food selectivity comes at a price: heterotrophic prokaryotes will need to hydrolyze most of those molecules into subunits small enough to be incorporated, because most molecules need to be smaller than 600 Da to pass through the prokaryotic cell wall (Weiss et al., 1991). For that purpose they use extracellular enzymes; so due to the central role of those enzymes they are referred to as the ‘gatekeepers’ of the C cycle (Arnosti, 2011). However, an alternative polysaccharide uptake mechanism of bacteria was recently revealed, which allows them to directly incorporate large molecular weight DOM compounds (Cuskin et al., 2015; Reintjes et al., 2017). Yet, not all high molecular weight DOM can be transported by this mechanism (i.e., biochemical and microbiological studies suggest that the binding/hydrolysis/transport is very selective for specific polysaccharides), and that mechanism still involve extracellular hydrolysis – but the binding proteins hang onto the pieces, such that they are transported into the cell with no loss to the external environment (Cuskin et al., 2015). Nevertheless, extracellular enzymatic activities (EEAs) are found from epipelagic to bathypelagic waters, commonly observing an increasing ratio of EEA to cell abundance with depth (Hoppe and Ullrich, 1999; Hoppe et al., 2002; Baltar et al., 2009).

## The Living and the ‘Living Dead’: Cell-Attached versus Cell-Free EEA

**FIGURE 1:**
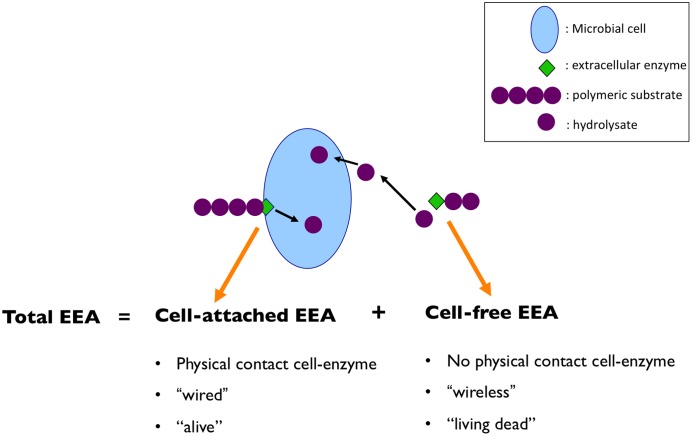
Total extracellular enzymatic activity (EEA) is the result of the combination of cell-associated and cell-free enzymes. Using the analogy of internet connection; cell-bound EEA would be the equivalent of a “wired” connection, whereas cell-free EEA would be a “wireless” connection. Moreover, the cell-bound EEA will be closely linked to the living cell’s behavior, whereas the cell-free enzymes will not necessarily be related to the factors affecting the microbial cells that originally produced them, and will remain active for a prolonged period of time, away from the cell. Thus, using another analogy, we could say that the cell-attached EEA would be more closely related to an “alive” activity, whereas cell-free EEA would be in the “living dead” realm.

Extracellular enzymes exist in two forms; cell-bound (i.e., cell-attached), and dissolved (i.e., cell-free, operationally defined as passing through a 0.22 μm filter). The total EEA is the result of the combination of cell-associated and cell-free enzymes (**Figure 1**). Using the Internet as an analogy, we could say that cell-bound EEA would be the equivalent of a “wired” Internet connection, whereas cell-free EEA would be a “wireless” connection (i.e., would still provide the end product – data transfer/hydrolysate – even if not physically connected to the hardware/cell). Different sources of cell-free enzymes have been suggested, including the active release by bacteria in response to an appropriate substrate (Alderkamp et al., 2007) and bacterial starvation (Albertson et al., 1990), and changes in cell permeability (Chrost, 1990). These studies indicate that marine bacteria can release enzymes into the environment not only to facilitate the hydrolysis of specific substrates during exponential growth on specific substrates, but also that starved cells during stationary phase showed a greater release of extracellular enzymes than at the onset of starvation. In fact, even during this starvation period, *de novo* protein synthesis occurs for the production and/or release of the cell-free enzymes into the surrounding environment, highlighting the importance of this process for some marine bacteria (Albertson et al., 1990). Besides direct release, there are other indirect sources of cell-free enzymes, including grazing on bacterial communities (Bochdansky et al., 1995), and viral lysis (Karner and Rassoulzadegan, 1995).

The question then is whether the strategy of releasing enzymes into the aquatic environment is energetically and ecologically efficient. Extracellular enzymes released by bacteria have been shown to produce sufficient products from particulate organic carbon to support the growth of the bacteria in the absence of any other significant source of organic carbon and without direct contact between the cell and particulate substrate (Vetter and Deming, 1999). These empirical results obtained by Vetter and Deming (1999) support the predictions they formulated in a model (Vetter et al., 1998), suggesting that dissolved extracellular enzymes are advantageous when bacteria are attached to particles and when the substrate is within a well-defined distance from the enzyme source. Furthermore, other model simulations later revealed that when enzyme-producing microbes competed with non-producers: (1) non-producers were favored by higher enzyme costs, (2) producers were favored by lower rates of enzyme diffusion, and (3) non-producers and producers coexisted in highly organized spatial patterns at intermediate enzyme costs and diffusion rates (Allison, 2005). These studies are also in agreement with the recently proposed solutions to the ‘public good dilemma’ in bacterial biofilms (Drescher et al., 2014). In that study, the secretion of public goods (i.e., chitinases) that potentially could be exploited by non-producing cells was explained by two mechanisms: cells can produce thick biofilms that confine the goods to producers, or fluid flow can remove soluble products of chitin digestion, denying access to non-producers. More recently, another model suggested that not only might cell-free enzymes be a profitable strategy to microbes live near or attached to particles, but went further to indicate that cell-free enzymes might be a viable strategy even for free-living cells, if substrate utilization is viewed as a cooperative effort (Traving et al., 2015). These authors also suggested that even small amounts of long-lived cell-free enzymes released by cell-free microbes could contribute significantly to the dissolved enzyme activity pool.

Once these enzymes are free, they can retain activity (i.e., are able to carry out their function) until they reach a specific substrate and then hydrolyze it. There are not many studies looking at the lifetime of these cell-free enzymes in seawater, but the ones available suggest that they can have half-life times of up to 20 days (Ziervogel et al., 2010; Steen and Arnosti, 2011; Baltar et al., 2013), and that deep water enzymes seem to have longer lifetimes than surface ones (Baltar et al., 2013). These observations are especially interesting because although it was originally thought that only cell-bound activity would be relevant in the marine environment (Rego et al., 1985; Chrost and Rai, 1993), evidence have been accumulating clearly indicating that the fraction of dissolved EEA is comparable to the bound fraction (Karner and Rassoulzadegan, 1995; Li et al., 1998; Baltar et al., 2010, 2013, 2016; Duhamel et al., 2010; Allison et al., 2012). In fact, in many cases, it has been observed that the proportion of dissolved EEA can reach a value of 100%, meaning that all the enzymatic activity, in those waters at the time of sampling, was being performed by cell-free enzymes (e.g., Karner and Rassoulzadegan, 1995; Baltar et al., 2010, 2016). Thus, a disconnect between rates of EEA and community composition is not surprising (D’ambrosio et al., 2014). When cell-free enzymes are responsible for a high proportion of the total EEA, a disconnection is produced between the microbes and the enzymatic activities; that is, a decoupling of *in situ* hydrolysis rates from actual microbial dynamics. Thus, a high proportion of dissolved EEA could indicate a greater importance of the history of the water mass than of the actual processes occurring at the time of sampling (Karner and Rassoulzadegan, 1995; Baltar et al., 2010, 2016; Arnosti, 2011).

Potential differences in temporal scales of activity for cell-attached and cell-free enzymes raises the question about controls on these pools of enzymes. The cell-bound EEA will be closely linked to the living cell’s behavior (i.e., be affected by the same factors controlling the growth, activity and diversity of living cells). In contrast, the cell-free enzymes will not necessarily be affected by the same factors influencing the cells, but will remain active for a prolonged period of time, and probably be affected by different factors (or in a different way in response to the same factors) than the cell-bound EEA. Bearing this in mind, using another analogy, we could say that the cell-bound EEA is an activity of the “living,” since that activity is performed attached to a cell which is alive, and thereby that activity will change in response to the cell’s needs and in response to the cells/community dynamics (**Figure 1**). Whereas the cell-free EEA is closer to an activity of the “living dead,” in the sense that it is not “alive” anymore because it is separated from the living cell, but still remains in some way “alive” in the sense that it can still perform its function when it encounters the right substrate.

However, it is important to realize that particle encounter is not necessarily the end of the line for an enzyme, and a large fraction of the cell-free enzymes may be trapped by particles including colloids or liposomes. In fact, the lifetime of cell-free enzymes can be extended if they are associated with particles (Gianfreda and Scarfi, 1991; Ziervogel et al., 2007), since surface associations can offer an improved resistance to physicochemical degradation (Lähdesmäki and Piispanen, 1992), and protection from remineralization (Lozzi et al., 2001). Evidence of bacterial cell-free enzymes embedded in an exopolymeric matrix have been reported (Decho, 1990), where cell-free EE attached to this matrix can form a complex similar to the enzyme–humic complexes in soils (Chrost, 1990). Also, some enzymes might be associated with particles due to trapping of digestive enzymes within partially degraded bacterial membranes which act as micelles (liposomes) (Nagata and Kirchman, 1992).

## Fate of the ‘Living Dead’ Cell-Free EEA

The activities of extracellular enzymes control the rate at which organic matter is processed in the ocean. Given the evidence of high activities of cell-free enzymes, understanding the controls on the lifetimes and activities of these cell-free enzymes is essential.

There are very few studies on this topic but they are already starting to reveal some of the key factors controlling the relative importance of cell-free EEA, and temperature seems to be a major one. In a Baltic Sea seasonal (1.5 y-long) study, a significant inverse relation was found between the proportion of dissolved relative to total EEA (Baltar et al., 2016). In a lab experiment, incubating microbial communities from the Great Barrier Reef waters (Australia) at three different temperatures (i.e., *in situ*, +3 and -3°C), a significant inverse relation was found again between temperature and the relative proportion of dissolved to total EEA (Baltar et al., 2017). These results are consistent with the increased observed in the proportion of dissolved relative to total EEA with depth as the temperature drops along the whole water column in a (sub) tropical Atlantic Ocean transect (Baltar et al., 2010). These studies signpost, from different angles (i.e., seasonal, climate change lab, and transect cruise studies), temperature as the main factor affecting the relative importance and activity of cell-free EEA. All those studies seem to indicate that the warmer the temperature the lower the proportion of dissolved EEA. This is also consistent with the longer lifetime of cell-free enzymes found for deep relative to the surface waters (Baltar et al., 2013).

Another factor that seems to be significantly contributing to controlling the lifetimes of cell-free enzymes is ultraviolet radiation (UVR). Very few studies are available on this topic too. The effect of UVR on cell-free enzymes directly was tested in Arctic seawater, finding that although natural illumination did not produce significant effects of photodegradation, a reduction in cell-free enzyme activity was found at artificially high UVR doses (i.e., UV-B intensity 5–10 times higher than *in situ*) (Steen and Arnosti, 2011). Interestingly, these authors found a significant effect of UVR on leucine aminopeptidase and alkaline phosphatase but not on beta-glucosidase at any treatment level. A recent study with cell-free enzymes from New Zealand waters revealed that environmentally relevant UVR irradiances reduced cell-free enzyme activities up to 87% in 36 h when compared to dark controls, likely a consequence of photodegradation (Thomson et al., 2017). This study also revealed that the magnitude of the effect of UVR on cell-free enzymes varied depending on the UVR fraction. Interestingly, consistent with the findings from Steen and Arnosti (2011), the effect of UVR differed depending on the enzyme; significantly decreasing the activity of cell-free leucine aminopeptidase and alkaline phosphatase, but not affecting β-glucosidase. This indicates that UVR (at ambient levels of radiation) can be a key factor reducing the activity (and lifetime) of cell-free enzymes (Thomson et al., 2017). Also, the fact that UVR effects vary among different enzymes indicates that UVR might change the spectrum of the EEA and thereby the composition of the resulting organic matter pool. Moreover, this effect of UVR on the cell-free EEA might help explain why the proportion of dissolved EEA tends to be lower in surface waters, and why the proportion of cell-free EEA is higher in winter and lower in summer.

More research is needed to fully constrain the factors and the mechanisms controlling the activity and lifetime of cell-free EEA as other factors, like pH, might be relevant. Moreover, the activities measured using externally-added substrates such as Leucine-MCA (7-amido-4-methylcoumarin) or MUF (4-Methylumbelliferyl)-β-glucose as substrates, reflect the activity of an unknown number/type of different enzymes that cleave the same substrate (Steen et al., 2015). Furthermore, enzymes of different primary and tertiary structure may hydrolyze the same substrate, but these enzymes would likely be susceptible to different degrees to UV radiation, heat inactivation, etc. Nevertheless, based on the evidence available thus far, and bearing in mind the projected warming ocean environment and the variable UVR light regime, it seems like there could be major changes in the activity of cell-free EEA and their contribution to organic matter remineralization in the near future.

## Conclusion and Future Outlook

It is clear now that, in any given marine location at any given time, cell-free EEA can be at least as important as cell-attached EEA. This has some strong implications on how we look and interpret many of the microbial parameters we measure since it can imply a decoupling between the community composition/function and the actual hydrolysis rates we measure. For instance, this complicates the study of functional redundancy in the marine environment if based on these extracellular enzymes, since changes in the microbial community composition might happen at a different temporal scale to the changes in EEA. Thus, it is important to consider the need to determine the cell-free fraction of EEAs (and not only the total fraction), if our aim is to link EEA to other microbial parameters.

In the near future, the advance in technology (e.g., gene- and protein-based techniques as well as organic matter characterization tools, etc.) will allow for a deeper understanding of the function and fate of cell-free EEA. For example, abundant periplasmic proteins were recently discovered using metaproteomics on seawater concentrates of the cell-free fraction (Xie et al., 2017). Based on that, these authors suggested that free proteins released from microbes could be important to ecosystem function (Xie et al., 2017). They admitted not having a clear explanation for the high presence of periplasmic proteins in the “non-bacterial” world. But, having in mind what has been discussed in this article, it is possible that many of those so-called “non-bacterial world” proteins by Xie et al. (2017) could indeed be cell-free (here described as “living dead”) extracellular enzymes. This is an example of how the use of novel technologies can help to make new discoveries in this field. However, we should not only focus on the development of new technologies but also complement those with the use of classical rate measurements. A more refined characterization of the DOM pool (e.g., LC–MS/MS) coupled with a combined determination of hydrolysis rates and gene/protein expression might open new avenues and discoveries in this field of research. Furthermore, these kinds of experiments, performed under different anthropogenic stressors, might also help elucidate how the role of these enzymes might change in response to different climatic scenarios. These studies will probably confirm that the ‘engines’ of the marine biogeochemical cycles are not only the microbial cells but that there are other processes taking place away from cells which are also an important part of that engine.

To conclude, the findings discussed in this article advocate for the need to go beyond the “living things,” – and study not only how the living cells/organisms will respond to anthropogenic perturbations, but also how the “living dead” cell-free active molecules will, if we really aim to fully constrain the future of marine biogeochemical cycles.

## Author Contributions

The author confirms being the sole contributor of this work and approved it for publication.

## Conflict of Interest Statement

The author declares that the research was conducted in the absence of any commercial or financial relationships that could be construed as a potential conflict of interest.
